# Novel insights into the potential role of ion transport in sensory perception in *Acanthamoeba*

**DOI:** 10.1186/s13071-019-3785-0

**Published:** 2019-11-14

**Authors:** Ruqaiyyah Siddiqui, Stephen K. Roberts, Timothy Yu Yee Ong, Mohammad Ridwane Mungroo, Areeba Anwar, Naveed Ahmed Khan

**Affiliations:** 1 Department of Biology, Chemistry and Environmental Sciences, College of Arts and Sciences, American University of Sharjah, University City, Sharjah, United Arab Emirates; 2 Biomedical and Life Sciences, Lancaster University, Lancaster, UK; 3 Department of Biological Sciences, School of Science and Technology, Sunway University, Bandar Sunway, Malaysia

**Keywords:** *Acanthamoeba*, Excystation, Encystation, Ion transporters, Drug targets

## Abstract

**Background:**

*Acanthamoeba* is well known to produce a blinding keratitis and serious brain infection known as encephalitis. Effective treatment is problematic, and can continue up to a year, and even then, recurrence can ensue. Partly, this is due to the capability of vegetative amoebae to convert into resistant cysts. Cysts can persist in an inactive form for decades while retaining their pathogenicity. It is not clear how *Acanthamoeba* cysts monitor environmental changes, and determine favourable conditions leading to their emergence as viable trophozoites.

**Methods:**

The role of ion transporters in the encystation and excystation of *Acanthamoeba* remains unclear. Here, we investigated the role of sodium, potassium and calcium ion transporters as well as proton pump inhibitors on *A. castellanii* encystation and excystation and their effects on trophozoites.

**Results:**

Remarkably 3′,4′-dichlorobenzamil hydrochloride a sodium–calcium exchange inhibitor, completely abolished excystation of *Acanthamoeba.* Furthermore, lanthanum oxide and stevioside hydrate, both potassium transport inhibitors, resulted in the partial inhibition of *Acanthamoeba* excystation. Conversely, none of the ion transport inhibitors affected encystation or had any effects on *Acanthamoeba* trophozoites viability.

**Conclusions:**

The present study indicates that ion transporters are involved in sensory perception of *A. castellanii* suggesting their value as potential therapeutic targets to block cellular differentiation that presents a significant challenge in the successful prognosis of *Acanthamoeba* infections.

## Background

In the course of the past few decades *Acanthamoeba* have acquired much attention as important human pathogens producing vision-threatening keratitis and a rare but fatal encephalitis known as granulomatous amoebic encephalitis (GAE) [1, 2]. The most disturbing characteristic is that the mortality concomitant with GAE because of pathogenic *Acanthamoeba* has endured significance (more than 90%) in spite of developments in antimicrobial chemotherapy and supportive care. Likewise, present diagnosis of *Acanthamoeba* keratitis is difficult [1, 3], and the existing treatments are lengthy and not entirely effective against all strains [4], in part this is owing to the ability of amoebae to convert into resistant cysts [5, 6], occasioning in infection recurrence. Additionally, cysts can endure up to several years while maintaining their pathogenicity, and this poses a major problem in chemotherapeutic treatment [5–7]. Cysts are double-walled, comprising of an outer ectocyst and an inner endocyst. Both walls meet at points known as arms or rays. Moreover, the cysts comprise pores identified as “ostioles”, these are acknowledged by the presence of an operculum bounded by a circular ridge that is apparent on the surface of mature cysts [8]. Ostioles are most likely used to observe environmental changes. As long as conditions are harsh, amoebae remain in the cyst form. Under favourable conditions, amoebae emerge from the cyst form and reproduce, resulting in infection recurrence [9].

To date, much of the research in *Acanthamoeba* has been concentrated on the infective trophozoite form and its pathogenic mechanisms. For example, in a recent study an ATP-sensitive potassium channel in the mitochondria of *A. castellanii* trophozoites was described [10]. However, the cellular differentiation processes, as well as how the cysts monitor the environment remains incompletely understood. The main components of cyst wall are acid-resistant proteins and cellulose that provide a physical barrier and making it resistant to biocides, desiccation and drugs [11]. These findings suggested further that redox balance reactions and membrane functions are potential target for the rational development of therapeutic interactions [11]. For cellulose, glycogen phosphorylase is the key enzyme that breaks down glycogen to provide glucose for the synthesis of cellulose [11, 12]. Garajová et al. [13] detected clustering of intramembranous particles during encystation. It was suggested that during endocyst formation, intramembranous particle clusters represent cellulose microfibril terminal complexes involved in cellulose synthesis that are reduced after cyst wall completion. It was proposed that disruption of this pathway would affect the synthesis of cyst wall and cyst resistance to chemotherapeutic agents. Given lack of specific drug to target *Acanthamoeba* infections, in addition to cellulose, polysaccharides composed of galactose are also highlighted as potential targets [14]. This could prove to be an important target given that there is a lack of established drug targets for a pharmaceutical intervention at the cyst stage.

In the present study, for the first time we investigated the role of ion transporters in sensory perception in the cyst stage of *A. castellanii* belonging to the T4 genotype. Based on the genome information, the presence of potassium ion transporters has been suggested [15]. However, their functional role in phenotypic switching is missing. Furthermore, identification of the ion transport pathway(s) in the cyst stage may guide in the detection of new anti-amoebic targets, as it is the cyst stage which presents a key challenge in treatment. Here, we investigated the effects of potassium, sodium and calcium transporter and proton pump inhibitors in cellular differentiation of *A. castellanii* of the T4 genotype.

## Methods

### Ion transporter inhibitors

Ion transport inhibitors were purchased from Sigma-Aldrich (Subang Jaya, Malaysia and dissolved in the solvents dimethyl sulfoxide (DMSO), distilled water, or 95% ethanol. Table 1 depicts the chemical nomenclature, class, empirical formula, molar mass and solvent solubility of the ion transport inhibitors. Stock solutions were prepared at 5 mM concentration.

**Table 1:** The chemical nomenclature, class, empirical formula, molar mass and solvent of ion transport inhibitors tested against *A. castellanii*

Compound	Chemical composition^a^ (if applicable)	Class	Empirical formula	Molar mass	Solvent and solubility
Gadolunium (III) chloride anhydrous		Cation channel inhibitor	GdCl_3_	263.61	Water
Strontium chloride anhydrous		Calcium channel inhibitor	SrCl_2_	158.52	Ethanol
CLP257	(5Z)-5-[(4-Fluoro-2-hydroxyphenyl)methylene]-2-(tetrahydro-1-(2H)-pyridazinyl)-4(5H)-thiazolone	Potassium chloride cotransporter	C_14_H_14_FN_3_O_2_S	307.34	DMSO 20 mg/ml
Tenatoprazole	(RS)-3-Methoxy-8-[(4-methoxy-3,5-dimethyl-pyridin-2-yl)methylsulfinyl]-2,7,9-triazabicyclo[4.3.0]nona-2,4,8,10-tetraene	Proton pump inhibitor	C_16_H_18_N_4_O_3_S	346.40	DMSO 5 mg/ml
PF-03716556	PF-03716556, [N-(2-Hydroxyethyl)-N,2-dimethyl-8-{[(4R)-5-methyl-3,4-dihydro-2H-chromen-4-yl]amino}imidazo[1,2-a]pyridine-6-carboxamide]	Acid pump inhibitor	C_22_H_26_N_4_O_3_	394.47	DMSO ≥10 mg/ml
3′,4′-dichlorobenzamil hydrochloride	L-594,881	Sodium–calcium exchanger inhibitor	C_13_H_12_N_7_OCl_3_ HCl	425.10	DMSO 30 mg/ml
Stevioside hydrate	(4α-13-[(2-O-β-d-Glucopyranosyl-β-d-glucopyranosyl)oxy]kaur-16-en-18-oic acid β-d-glucopyranosyl ester	Potassium channel	C_38_H_60_O_18_ xH_2_O	804.87 (anhydrous basis)	DMSO 25 mg/ml
Cariporide	HOE-642, N-(Aminoiminomethyl)-4-(1-methylethyl)-3-(methylsulfonyl)-benzamide, N-(Diaminomethylene)-4-isopropyl-3-(methylsulfonyl)benzamide	Sodium–hydrogen exchanger inhibitor	C_12_H_17_N_3_O_3_S	283.35	DMSO 20 mg/ml
Lanthanum oxide		Potassium channel	La_2_O_3_	325.808	Dilute nitric acid

^a^If applicable

### Cultivation of *Acanthamoeba castellanii*

*Acanthamoeba castellanii* was acquired from American Type Culture Collection (ATCC) 50492 of the T4 genotype and grown axenically in 75 mm^3^ culture flasks at 30 °C in proteose peptone yeast glucose (PYG) medium (proteose peptone 0.75%, yeast extract 0.75%, glucose 1.5%) as described previously [16]. Growth media were refreshed every 20 h to obtain vegetative trophozoites. Flasks were examined under phase contrast microscope on a daily basis until confluent *A. castellanii* were observed prior to experimentation.

### Amoebicidal assays

To study the role of ion transport inhibitors on the viability of *A. castellanii* trophozoites, 5 × 10^5^ amoebae/0.5 ml/well were dispersed in Roswell Park Memorial Institute medium (RPMI-1640) in the absence or presence of various inhibitors at different concentrations as described previously [17]. Plates were incubated at 37 °C for 24 h. Subsequently, viability of the amoebae was determined by addition of 0.1% Trypan blue and the number of living (non-stained) and dead (stained) *A. castellanii* counted by means of a haemocytometer. Control experiments were conducted in RPMI-1640 alone and RPMI-1640 containing respective amount of solvents. The data are representative of three independent experiments and are given as the mean ± standard error.

### Amoebistatic assays

An amoebistatic assay was employed to establish the role of ion transport inhibitors on *A. castellanii* growth [1]. Specifically, 5 × 10^5^ trophozoites were incubated with varying concentrations of the inhibitors in growth medium, i.e. PYG in 24-well plates. Plates were reserved at 30 °C for 48 h. As controls, 5 × 10^5^ trophozoites were inoculated in 100% PYG medium, 100% non-nutritive phosphate-buffered saline (PBS) solution and the corresponding amounts of solvents plus PYG medium, and kept in the aforementioned conditions. Subsequently, the number of amoebae was quantified *via* haemocytometer enumeration. All experimental data are representative of the mean ± standard error of three experiments conducted in duplicate.

### Provision of *A. castellanii* cysts and excystation assays

To acquire *A. castellanii* cysts, encystation was motivated by dispersing 5 × 10^6^ *A. castellanii* trophozoites on non-nutrient agar plates. Plates were kept at 30 °C for 14 days [16, 18]. Food deprivation ensued in conversion from the trophozoite stage into the cyst form. Afterward, 10 ml of PBS was incorporated to each plate. Cysts were scratched off the agar surface with a cell scraper and counted using a haemocytometer. To study effects of ion channel inhibitors on excystation, assays were carried out by placing *A. castellanii* cysts in the presence or absence of different concentrations of inhibitors in PYG medium (2 × 10^4^ cysts per ml per well of 24-well plates). Plates were kept at 30 °C and observed every 24 h for the appearance of viable trophozoites for up to 72 h.

### Encystation assays

To determine the effects of inhibitors on cyst formation, amoebae inoculation on agar plates is required. For this purpose, encystation assay using liquid medium was used as described previously [19]. In brief, 2 × 10^6^ amoebae were dispersed in PBS and 50 mM MgCl_2_ and 10% glucose (i.e. encystation trigger) in a 24-well tissue culture plates at 30 °C for 72 h (without shaking). Following incubation, SDS (0.5% final concentration) was added for 10 min to solubilize trophozoites and the cysts were counted using a haemocytometer. To study effects of ion channel inhibitors on encystation, assays (PBS plus 50 mM MgCl_2_ and 10% glucose) were conducted in the presence of varying concentrations of the inhibitors. In brief, 2 × 10^6^ amoebae were dispersed in PBS containing varying concentrations of inhibitors and in the presence of 50 mM MgCl_2_ and kept at room temperature for 20 min. Next, 10% glucose was added as a trigger for encystation and plates were incubated at 30 °C for 72 h. Encystation in wells without inhibitors was used as positive controls and wells without the inhibitors as well as encystation trigger were used as negative controls. The corresponding amounts of solvents were incubated in wells plus *A. castellanii* and used as solvent controls. Amoebae were counted using a haemocytometer [20]. All experimental data are representative of the mean ± standard error of at least three independent experiments completed in duplicate.

### Statistical analysis

Statistical significance for differences was evaluated using a 2-sample t-test; two-tailed distribution, comparing the mean and resulting *P*-values < 0.05 were considered significant. For graphical representation of the data, y-axis error indicates the standard error of the data.

## Results

### 3′,4′-Dichlorobenzamil hydrochloride, stevioside hydrate and CLP257 inhibited excystation of *A. castellanii*

**Fig. 1:**
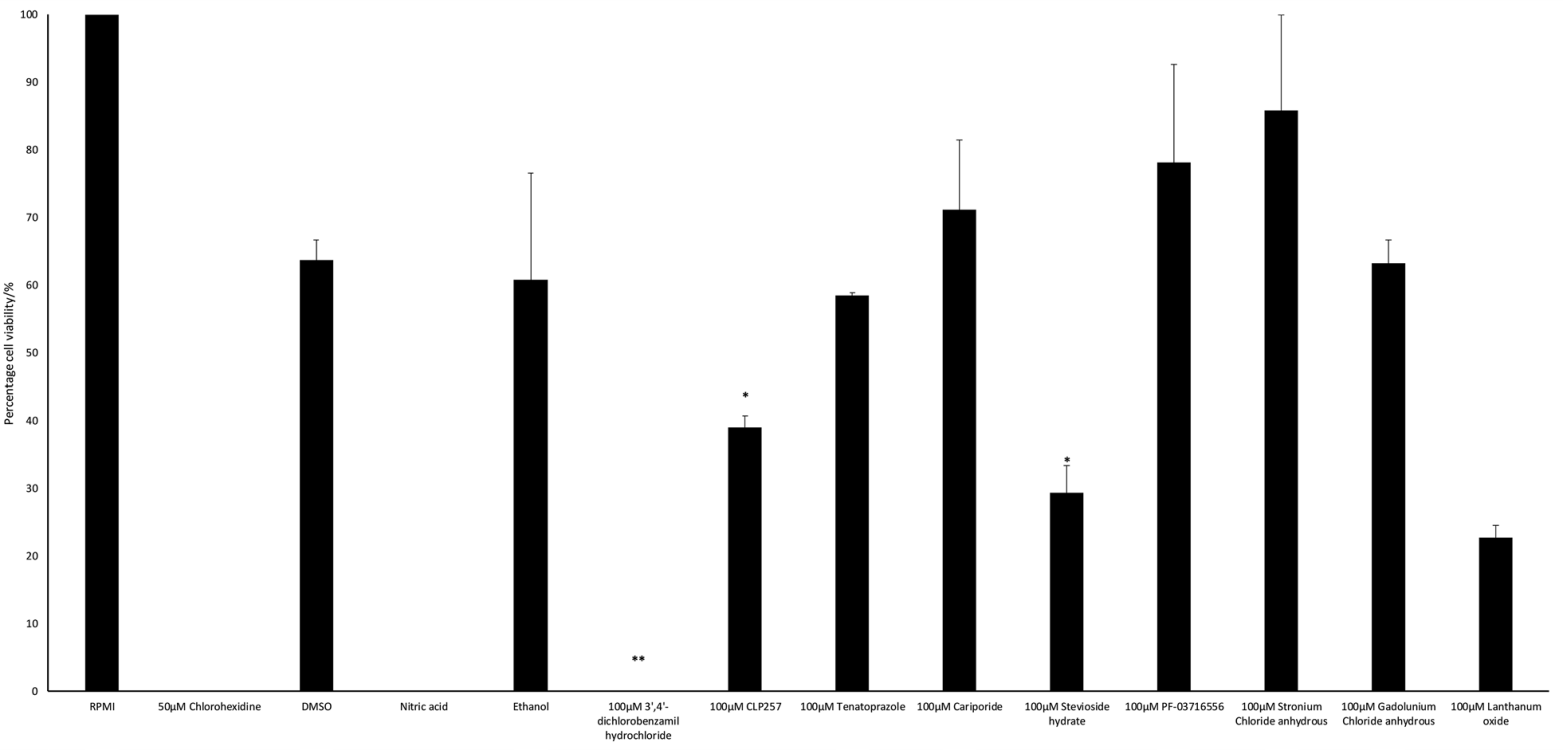
Percentage of survived amoebae from cyst transformation to amoeboid form after 24 hours of incubation in PYG medium at 30 °C. 100 μM strontium chloride preserved the ability of amoeboid transformation as the survival reached 85.78%; similarly 100 μM PF-03716556 resulted in viability of 78.15%. 100 μM cariporide treatment resulted in 71.13% viable converted trophozoites. On the other spectrum of viability, treatment with 100 μM 3′,4′-dichlorobenzamil hydrochloride completely inhibited the excystation process. The use of 100 μM lanthanum oxide and stevioside hydrate resulted in a partial inhibition of the excystation, with respective cell viability of 22.75% and 29.41%, respectively. Asterisks represent significance of differences in comparisons with controls (*P < 0.05, **P < 0.01). The figure is representative of three experiments

To examine the effects of ion transporter inhibitors on the excystation of *A. castellanii* cysts, excystation in amoebae incubated without inhibitors was considered as 100% and the effects of inhibitors/solvent are presented as the relative change. Of note, 100 μM 3′,4′-dichlorobenzamil hydrochloride, a sodium–calcium exchange inhibitor, abolished the excystation of *A. castellanii* as depicted in Fig. 1. Moreover, this result was statistically significant when compared to the solvent control (DMSO) (mean ± SD, 63.73 ± 4.16) (*t*_(1)_ = 21.67, *P* = 0.032). In addition, 100 μM stevioside hydrate a potassium transport inhibitor and CLP257, a potassium chloride transport inhibitor, resulted in 29.41% excystation and 39% excystation compared with 100% excystation in untreated amoebae. Both results (mean ± SD, 29.41 ± 5.55 and 39 ± 2.46) were statistically significant when compared to the solvent control (DMSO) (63.73 ± 4.16) (*t*_(2)_ = 7.00, *P* = 0.024 and *t*_(2)_ = 7.23, *P* = 0.02). Gadolunium (III) chloride (100 μM), also resulted in inhibition of the excystation of *A. castellanii* resulting in 63.24% emerging trophozoites. On the contrary, the use of 100 μM lanthanum oxide resulted in the inhibition of the excystation by 77.25% when compared to the untreated amoebae but effects were not statistically significant when compared to the solvent control (nitric acid). Likewise, 100 μM of tenatoprazole (proton pump inhibitor), cariporide (Na^+^/H^+^ exchange inhibitor), PF-03716556 (acid pump antagonist) and strontium chloride (interact with ligands that normally bind calcium) resulted in 58.42%, 71.13%, 78.16% and 85.78% emerging trophozoites, respectively, and the results were not statistically significant when compared with the solvent controls. To further confirm these findings, another *A. castellanii* (50494 strain) was used. When tested against the 50494 strain, the results were consistent with the 50492 strain and revealed that among various inhibitors tested, 3′,4′-dichlorobenzamil hydrochloride, stevioside hydrate and CLP257 showed inhibition of excystation. In summary, the results revealed that 3′,4′-dichlorobenzamil hydrochloride, stevioside hydrate and CLP257 inhibited excystation of *A. castellanii*.

### Ion transport inhibitors did not inhibit the encystation of *A. castellanii*

**Fig. 2:**
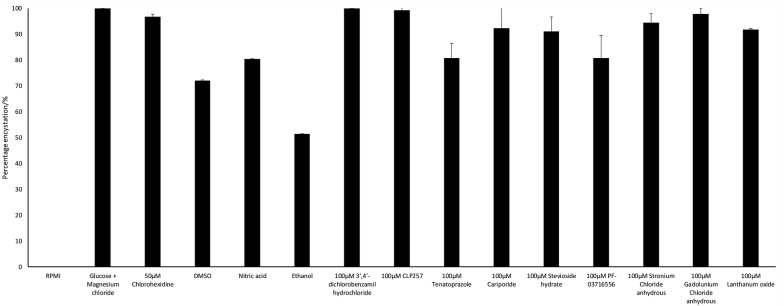
Percentage of amoebae undergoing encystation after the treatment with ion transport inhibitors in encystation medium (50 mM MgCl_2_ and 10% glucose dissolved in PBS). 100 μM PF-03716556 and tenatoprazole resulted in 80.73% and 80.77% of cells undergoing cyst transformation, respectively, which is the lowest among the ion channel blockers. The figure is representative of three experiments

To determine the effects of ion transport inhibitors on the encystation of *A. castellanii*, encystation assays were carried out. Untreated amoebae showed 0% encystation. Treatment with 100 μM PF-03716556, tenatoprazole, stevioside hydrate, lanthanum oxide, cariporide, strontium chloride, gadolunium (III) chloride, CLP257 and 3′,4′-dichlorobenzamil hydrochloride resulted in 80.73%, 80.77%, 91.16%, 91.81%, 92.32%, 94.34%, 97.87%, 99.34% and 100% encystation, respectively, as depicted in Fig. 2. However, these results were not different when compared with the respective solvent controls.

### Ion transport inhibitors did not exhibit amoebicidal activity against *A. castellanii* trophozoites

**Fig. 3:**
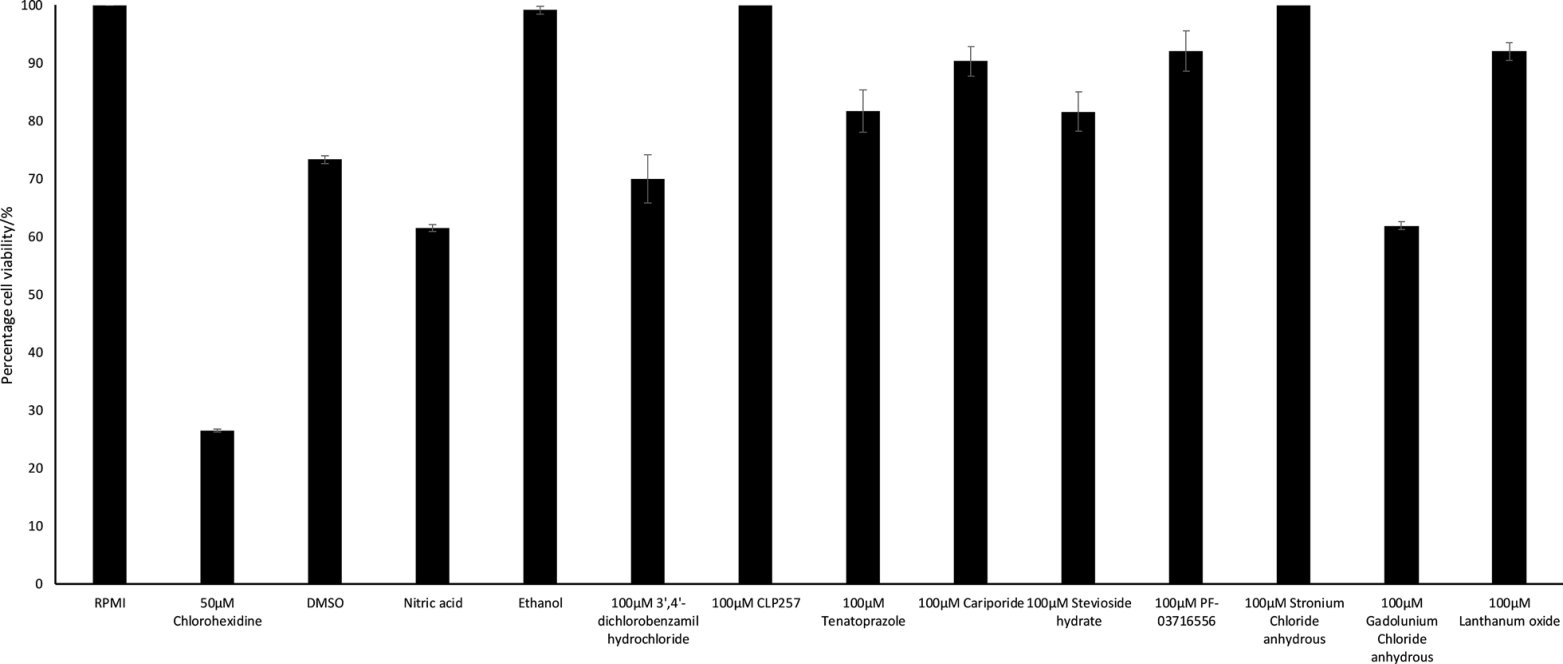
Percentage of viable cells after *A. castellanii* trophozoites were incubated with the ion transport inhibitors in RPMI 1640 in 96-well culture plates for 24 hours at 30 °C. The ion channel inhibitors did not show amoebicidal effects against *A. castellanii*. The figure is representative of three experiments

To assess the effects of ion transport inhibitors, amoebicidal assays were accomplished. Viability in untreated amoebae was considered as 100% and the effects of inhibitors/solvent are presented as the relative change. The results showed that ion transport inhibitors had no amoebicidal activity against *A. castellanii* trophozoites (Fig. 3). In the presence of 100 µM 3′,4′-dichlorobenzamil hydrochloride, and gadolinium (III) chloride, the percentage cell viability was 70% and 61.92%, respectively. However, these results were not different when compared with the respective solvent controls. The use of 100 μM of stevioside hydrate, tenatoprazole, cariporide, PF-03716556, lanthanum oxide, CLP257 and strontium chloride did not reveal any statistically significant amoebicidal activity as they resulted in respective cell viability of 81.62%, 81.73%, 90.3%, 92.03%, 92.05%, 100% and 100%. Overall, the results revealed that the ion transport inhibitors had limited or no effects on the viability of *A. castellanii* trophozoites.

### Ion transport inhibitors did not inhibit the growth of *A. castellanii* trophozoites

**Fig. 4:**
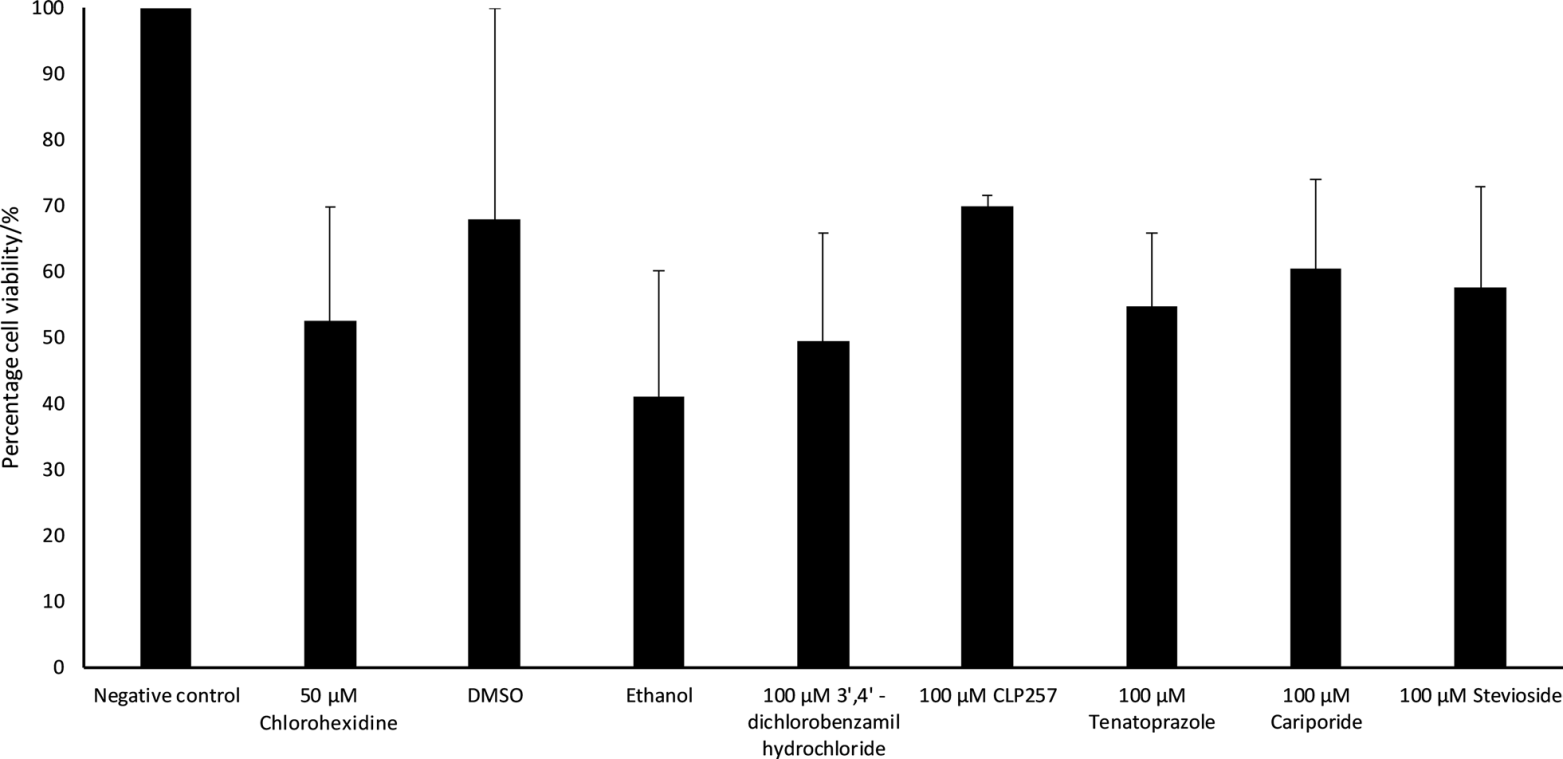
Percentage of viable cells after *A. castellanii* were incubated with the ion channels in PYG for 24 hours at 30 °C, the ion transport inhibitors had not shown inhibitory action against the growth of *A. castellanii* as the viability ranged between 40–60% survival. The figure is representative of three experiments

Amoebistatic assays were completed in the presence and absence of ion transport inhibitors to study the effects of the inhibitors on the growth of *A. castellanii* trophozoites. Growth in untreated amoebae was considered as 100% and the effects of inhibitors/solvent are presented as the relative change. The number of untreated amoebae increased from 5 × 10^5^ to 1.17 × 10^6^ when incubated in PYG growth medium. Addition of 100 μM of strontium chloride, PF-03716556, 3′,4′-dichlorobenzamil hydrochloride, tenatoprazole, gadolinium (III) chloride, stevioside hydrate, cariporide, CLP257 resulted in percentage growth of 44.55%, 45.80%, 49.42%, 54.62%, 55.45%, 57.58%, 60.52% and 69.81%, respectively, as shown in Fig. 4. However, none of the growth inhibition was statistically significant when compared with the corresponding solvent controls.

## Discussion

Membrane ion transporters are well known to be pertinent to the permeability of ions, membrane potential and stability of cell microenvironment ranging from single cells to large complex organisms. Microbes possess a considerable selection of ion transporters in their cell membranes [21]. Cellular functions in microbes require tight regulation and it is well accepted that ion channels have a crucial role, for instance in energy transduction [22]. For example, potassium channels are one of the most varied families of membrane proteins, widely described from bacteria to humans. They are found in all eukaryotic organisms, marking them out as essential biological enzymes. Their known roles comprise voltage-membrane potential maintenance, excitability, organogenesis, cell death, pH and cell volume regulation. They are involved in sensing and responding to environmental changes such as acidification, oxygen pressure, osmolarity and ionic concentration [23].

It is not clear how *Acanthamoeba* cysts monitor environmental changes and determine favourable conditions leading to their emergence as viable trophozoites. There is limited understanding regarding sensory perception in the cyst stage of *Acanthamoeba*.

Cyst walls are largely composed of acid-resistant proteins and polysaccharides, among which cellulose is one of the key constituents [13]. Magistrado-Coxen et al. [24] demonstrated that purified cyst walls retained an outer ectocyst layer, an inner endocyst layer, and ostioles that connect them. The cyst wall proteins were mostly represented by three families of lectins and are localized in the ectocyst, the endocyst layer and ostioles of mature walls [24], suggesting their potential role in monitoring the environmental conditions.

In this study, for the first time we postulated that ion transport plays a role in sensory perception in the cyst stage of *A. castellanii.* Our experiments showed that tenatoprazole, a proton pump inhibitor, partially inhibited the formation of cysts while hampering the excystation process to a greater extent. This suggests that proton pump may be involved in the conversion of cysts into trophozoites. Moreover, previous studies in *Dictyostelium discoideum* revealed that the plasma membrane proton pump served as an intercellular pH regulator [25]. It is plausible that the proton may regulate the pH so as to detect when there are favourable conditions for the trophozoite to emerge, hence being important for excystation. In addition, cariporide, a sodium–proton exchanger, was found to partially inhibit excystation. Sodium hydrogen ion exchange is likely to be involved in regulating pH, and acting similarly to tenatoprazole for trophozoite formation in *Acanthamoeba*. Furthermore, both stevioside and lanthanum oxide which are potassium channel modulators of activity completely inhibited the excystation process and yet did not have much effect in the transformation of the trophozoites into cyst, indicating that potassium transport may be involved in the sensory perception of the cyst to emerge as a trophozoite but not *vice versa*. Similarly, CLP257, a potassium-chloride co-transporter activator, also inhibited the excystation to some extent, again suggesting the role of potassium ion transport in the excystation process of *A. castellanii*. Notably, 3′,4′-dichlorobenzamil hydrochloride, a sodium–calcium exchanger, completely abolished excystation of *A. castellanii*. This indicates that the Ca^2+^ signalling is disturbed and leads to phenotypic transformation. These findings are interesting and can be utilised as a potential drug target. To underline the importance of ion transport in drug discovery, 13% of all drugs on the market to date are targeting ion transporters; this makes them the second most important target for medical intervention after the G-protein coupled receptors [26].

Comparable to other microbes, *Acanthamoeba* has been shown to display chemosensory responses and is known to have receptor(s) in its plasma membranes to detect chemo-attractants [27]. It was previously suggested that there may be specific receptors that can be comparable to sensory organs for taste and smell to detect favourable prey and lead to subsequent motor mechanisms and determine *Acanthamoeba* preferential feeding behaviour towards certain bacterial species and it is plausible that ion channels are utilised in these processes [28].

## Conclusions

To our knowledge, we demonstrated for the first time that ion transport plays a role in sensory perception in the cyst stage of *A. castellanii* and could be valuable targets in the rational development of chemotherapeutic interventions. Subsequent studies will determine optimal ion transport activity and associated molecules, knowledge of which can be exploited for blocking excystment and improved strategies for therapeutic interventions. Moreover, identifying the genes encoding ion channels (and transporters) in *Acanthamoeba* and expression in a heterologous expression cell system will enable their electrophysiological characterisation using patch clamp techniques that can provide information on the functional aspects of important membrane proteins that maybe used as anti-amoebic targets for treatment of these important pathogens.

## Data Availability

All data generated or analysed during this study are included in this published article.

## Abbreviations

GAE: granulomatous amoebic encephalitis
DMSO: dimethyl sulfoxide
ATCC: American type culture collection
PYG: protease peptone yeast glucose medium
RPMI: Roswell Park Memorial Institute medium
PBS: Phosphate buffered saline
SDS: sodium dodecyl sulphate
